# The complete plastome sequence of the endangered orchid *Kuhlhasseltia nakaiana* (Orchidaceae)

**DOI:** 10.1080/23802359.2017.1390408

**Published:** 2017-10-14

**Authors:** Young-Kee Kim, Myoung Hai Kwak, Ja-Ram Hong, Hoe-Won Kim, Sangjin Jo, Jung-Yeon Sohn, Se-Hwan Cheon, Ki-Joong Kim

**Affiliations:** a Division of Life Sciences, Korea University, Seoul, Korea;; b Department of Plant Resources, National Institute of Biological Resources, Incheon, Korea

**Keywords:** Plastome, *Kuhlhasseltia nakaiana*, Orchidaceae, endangered species

## Abstract

In this study, we report the complete plastome sequence of *Kuhlhasseltia nakaiana* (F.Maek.) Ormerod (Orchidaceae) (NCBI acc. no. KY354041), an endangered plant species protected by the national law of Korea. The gene order and number in the *K. nakaiana* plastome were similar to a typical orchid plastome. The complete plastome was 147,614 bp in length and consisted of a large single copy region of 81,617 bp and a small single copy region of 13,673 bp, separated by two inverted repeats of 26,162 bp. The plastome contained 103 genes, of which 69 were protein-coding genes, 30 were tRNA genes, and four were rRNA genes. Fourteen genes contained one intron and two genes (*clp*P and *ycf*3) had two introns. The AT content of the plastome was 60.5%. A total of 74 simple sequence repeat regions were identified from the plastome. Phylogenetic analysis determined that *K. nakaiana* was a member of the tribe Cranichideae and revealed the sister group relationship between *K. nakaiana* and *Ludisia discolor* within the tribe Cranichideae.

*Kuhlhasseltia nakaiana* (F.Maek.) Ormerod, a terrestrial orchid in the genus *Kuhlhasseltia*, is native to Korea, Japan, Taiwan, and the Philippines (Saeki et al. 2014). The genus *Kuhlhasseltia* is composed of five species (Lee 2011). In nature, *K. nakaiana* is very rare and only three populations were recorded in Korea. This species has, therefore, been designated an endangered and protected plant species in Korea. It is a tiny plant of less than 10 cm in height with only a few small leaves along a single erect stem. *K. nakaiana* belongs to the subfamily Orchidoideae of the family Orchidaceae (APG IV 2016). To develop genetic markers for *K. nakaiana* for use in conservation studies, we sequenced and analysed the plastome of this species.

*K. nakaiana* plant material was collected from its natural habitat of Jeju Island, Korea, under a collection permit from the environmental protection authority of the Korean government. As this species is seriously threatened species, we were permitted to collect only one *K. nakaiana* individual. Therefore, a representative specimen was not deposited in herbarium. But, the extracted DNAs were deposited in the Plant DNA Bank in Korea (PDBK 2016-0443). Fresh leaves were ground into powder in liquid nitrogen and total DNA was extracted using a G-spin™II Plant Genomic DNA extraction kit (iNtRON). The complete plastome sequence was generated using Illumina MiSeq (San Diego, CA) and assembled by Geneious 6.1.8 (Kearse et al. 2012). An average sequence coverage of 400 times the plastome size was obtained. Annotations were performed using the National Center for Biotechnology Information (NCBI) BLAST and tRNAscan-SE programs (Lowe and Eddy 1997). The complete plastome sequence was submitted to the NCBI database under the accession number KY354041.

The gene order and number in *K. nakaiana* were similar to those of a typical angiosperm such as *Panax*, *Nicotiana*, and *Sesamum* (Shinozaki et al. 1986; Kim and Lee 2004; Yi and Kim 2012), with the exception of the *ndh* genes. A typical plant plastome contains 11 *ndh* family genes; the *K. nakaiana* plastome, however, contained only the *ndh*E gene, with the other 10 *ndh* genes pseudogenized or lost. These *ndh* gene losses are not unique to the *K. nakaiana* plastome and occur commonly in the plastomes of Orchidaceae plants (Chang et al. 2006; Wu et al. 2010; Lin et al. 2015). In total, the plastome of *K. nakaiana* contained 103 unique genes including 69 protein-coding genes, 30 tRNA genes, and four rRNA genes. Fourteen genes had a single intron while the *clp*P and *ycf*3 genes had two introns. The length of the complete plastome of *K. nakaiana* was 147,614 bp; this was composed of a large single copy (LSC) region of 81,617 bp, a small single copy (SSC) region of 13,673 of bp, and two inverted repeats (IRs) of 26,162 bp. The average AT content of the plastome was 60.5%. We identified a total of 74 simple sequence repeat (SSR) loci including 61 mono-SSRs, 10 di-SSRs, and three tri-SSRs. Some of these plastome SSRs may useful for the development of genetic markers among *K. nakaiana* populations.

Phylogenetic analyses were performed on a dataset that included 78 protein-coding genes (excluding *ycf*1) and four rRNA genes extracted from 38 taxa in the NCBI database and *Cymbidium macrorhizon* (KY354040) and *K. nakaiana* (KY354041). *Fritillaria hupehensis* and *F. taipaiensis*, representing the sister order Liliales, were used as outgroups. The gaps for lost genes were treated as missing bases. The 82 gene sequences were aligned with MUSCLE in Geneious 6.1.8; the aligned data matrix consisted of a total of 70,620 bp. This alignment is used for phylogenetic analysis using RAxML v. 7.7.1 (Stamatakis et al. 2008). An ML tree was obtained with an ML estimation value of –302818.486357. The sister group relationship between *K. nakaiana* and *Ludisia discolor* was confirmed with 100% bootstrap value support (Figure 1). Both species occurred in the tribe Cranichideae within Orchidoideae.

**Figure 1.**
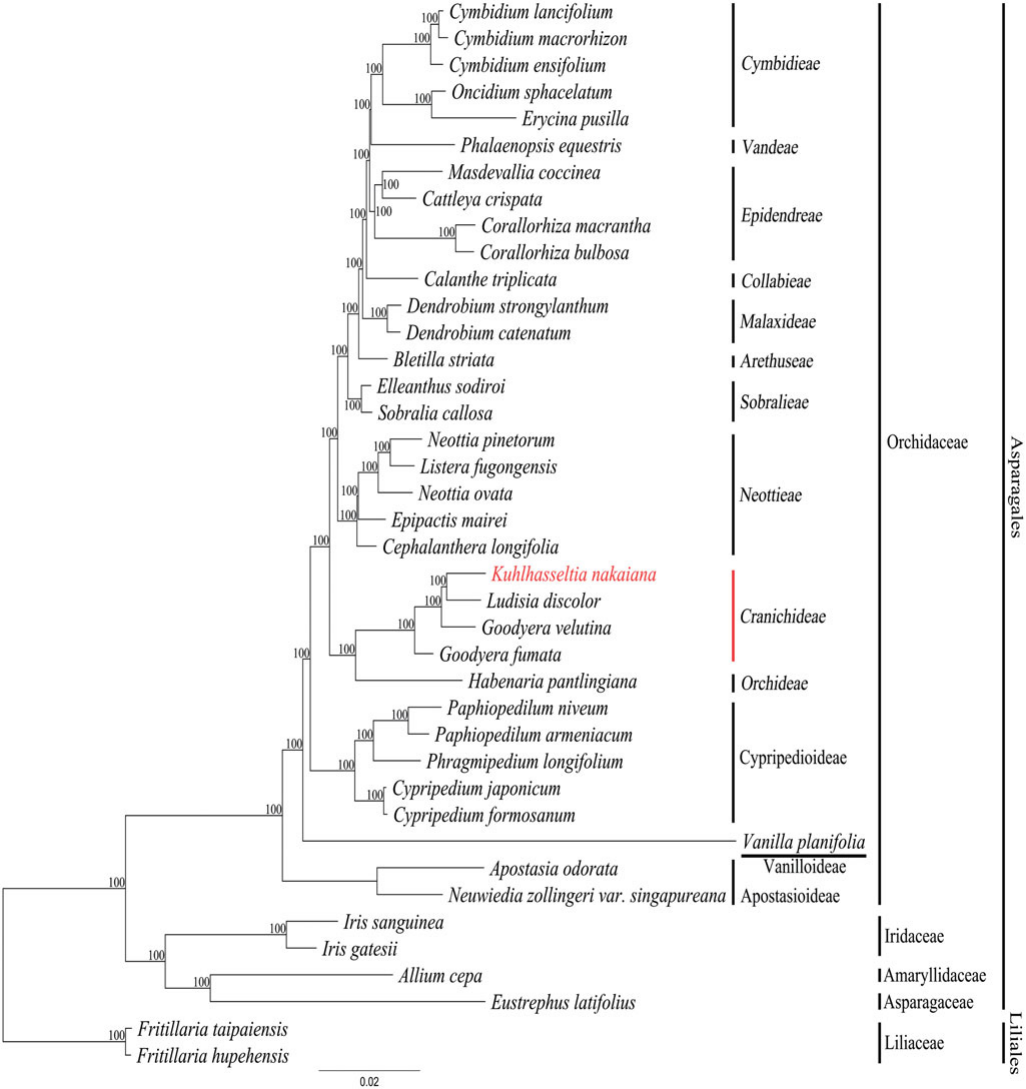
Chloroplast phylogenetic tree of Asparagales. A maximum likelihood tree (ML) inferred from analysis of alignment data containing 78 protein coding genes and four rRNA genes in 40 plastome sequences. The number above or below or each node indicates bootstrap value. Genbank accession numbers of taxa are shown below, *Allium cepa* (NC024813), *Apostasia odorata* (NC030722), *Bletilla striata* (NC028422), *Calanthe triplicata* (NC024544), *Cattleya crispata* (NC026568), *Cephalanthera longifolia* (NC030704), *Corallorhiza bulbosa* (NC025659), *Corallorhiza macrantha* (NC025660), *Cymbidium ensifolium* (NC028525), *Cymbidium lancifolium* (NC029712), *Cymbidium macrorhizon* (KY354040), *Cypripedium formosanum* (NC026772), *Cypripedium japonicum* (NC027227), *Dendrobium catenatum* (NC024019), *Dendrobium strongylanthum* (NC027691), *Elleanthus sodiroi* (NC027266), *Epipactis mairei* (NC030705), *Erycina pusilla* (NC018114), *Eustrephus latifolius* (NC025305), *Fritillaria hupehensis* (NC024736), *Fritillaria taipaiensis* (NC023247), *Goodyera fumata* (NC026773), *Goodyera velutina* (NC029365), *Habenaria pantlingiana* (NC026775), *Iris gatesii* (NC024936), *Iris sanguinea* (NC029227), *Kuhlhasseltia nakaiana* (KY354041), *Listera fugongensis* (NC030711), *Ludisia discolor* (NC030540), *Masdevallia coccinea* (NC026541), *Neottia ovata* (NC030712), *Neottia pinetorum* (NC030710), *Neuwiedia zollingeri var. singapureana* (KM244735), *Oncidium sphacelatum* (NC028148), *Paphiopedilum armeniacum* (NC026779), *Paphiopedilum niveum* (NC026776), *Phalaenopsis equestris* (NC017609), *Phragmipedium longifolium* (NC028149), *Sobralia callosa* (NC028147), *Vanilla planifolia* (NC026778).
